# Assessment of willingness of Saudi public to participate in a dental biorepository for research purposes

**DOI:** 10.1186/s12903-023-02775-9

**Published:** 2023-02-07

**Authors:** Aisha Mohammed Basudan, Lamis Khalid Dagriri, Ghaida Hamad Alnaqa, Joud Muhanna Alqahtani, Mohammad Ibrahim Alsowail

**Affiliations:** 1 College of Dentistry/King Saud bin Abdulaziz University for Health Sciences (KSAU-HS), Riyadh, Saudi Arabia. King Abdullah International Medical Research Center (KAIMRC), Riyadh, Saudi Arabia. Division of Orthodontics, Dental Services Department, King Abdulaziz Medical City (KAMC), Ministry of National Guard-Health Affairs (MNGHA), Riyadh, Saudi Arabia; 2 Intern, College of Dentistry, Riyadh Elm University, Riyadh, Saudi Arabia; 3 Intern, College of Dentistry, King Saud Bin Abdulaziz University of Health Sciences (KSAU-HS), Riyadh, Saudi Arabia

**Keywords:** Biobanks, Biorepositories, Dental biospecimens, Willingness to donate, Attitudes toward specimen donation, Knowledge about dental stem cells, Knowledge about biobanks

## Abstract

**Background:**

Biobanks/biorepositories are created to collect biospecimens for therapeutic and research uses. The success of the banking concept depends predominantly on the public's understanding and desire to contribute, which triggers several social, cultural, and ethical implications. The aim of this study is (1) to assess the willingness among adults attending outpatient clinics at King Abdulaziz Medical City to donate dental tissue samples to a biorepository for research purposes, (2) to identify the significant predictors for positive attitudes and willingness to donate dental bio-specimens.

**Methodology:**

This is a cross-sectional study that targeted 401 adult outpatients attending King Abdulaziz Medical City in Riyadh, Saudi Arabia. The questionnaire focused on three main parts: demographic and personal characteristics, and previous experience regarding biorepositories (part I), knowledge about dental biorepositories (part II), and willingness and attitudes towards donating dental biospecimens (part III). Data collected were analyzed using the statistical program SAS (version 9.4) with 0.05 level of significance to determine the willingness of donating tissue to biobanks for biomedical research purposes, measure knowledge and attitude about biobanking, find the association between the assessed variables, and identify significant predictors of positive attitude to donate dental biospecimens.

**Results:**

66% of the participants were willing to donate dental biospecimens, however only 33.9% showed good level of knowledge. Despite the notable lack of knowledge, 54% respondents had favorable attitude towards donating dental biospecimens, and only 17% were negative while the remaining 29% were neutral. Previous involvement in medical research, previous blood testing or donation, female gender, higher education level, employment in a medical facility, and higher monthly income variables were found to be significantly associated with higher willingness to donate dental biospecimens.

**Conclusion:**

Although the majority of the participants exhibited lack of knowledge about dental biorepositories, they showed high willingness and good attitude towards donating dental biospecimens. This favorable attitude is, in turn, encouraging for the future establishment of dental biorepositories in Saudi Arabia. Six factors were significantly associated with the willingness to donate dental biospecimens, out of these, female gender, previous blood testing/donation, previous involvement in medical research were found to be strong predictors.

**Supplementary Information:**

The online version contains supplementary material available at 10.1186/s12903-023-02775-9.

## Introduction

Biorepositories and/or biobanks are facilities that arrange and process biological specimens [1]. Collecting these biospecimens plays a major role in conducting biomedical research [2], in particular research fields related to tissue engineering, stem-cell regenerative therapy and precision medicine. In fact, biobanking can be a promising initiative for these fundamental research areas where biological samples, such as DNA, saliva, plasma, and tissue samples are collected and archived in combination with personal and clinical information of the donors [3]. In dentistry, biorepositories are needed to explore the links between oral/dental diseases and other systemic diseases [4] and provide personalized diagnostics/therapeutics by collecting mesenchymal stem cells (MSCs) that play a critical role in the regeneration of vital structures such as bone, cementum, periodontal ligaments, dental pulp, and neuronal cells [5].

As the evidence that dental tissues are rich sources of MSCs is continually growing, biobanks are now using discarded biological tissues such as extracted teeth (dental pulps), which are considered to be medical waste, to produce MSCs therapeutic batches [6–9]. Dental stem cells (DSCs), unlike embryonic stem cells, are accessible, highly reachable, and possess strong multipotency with fewer ethical considerations [9, 10], hence, several countries have placed dental biorepositories into practice. These stem cells have demonstrated therapeutic capability in both Oral Medicine and numerous fields of Medicine for tissue engineering regenerative therapy. It holds great potential to repair and regenerate bone, the central nervous system, liver tissue, heart tissue, eyes, muscles, salivary gland cells, and many other tissues [6, 8–11].

The success of biorepositories depends predominantly on the public's understanding, support, and their willingness to donate biospecimens; therefore, it is advisable to view them as biobank collaborators rather than participants [4, 12]. Several previous studies conducted have shown a positive attitude, high rate of willingness, and enthusiasm in the participation of donating dental biospecimens for biomedical research [4]. Italian and Swedish surveys revealed that 86% and 78% of the participants respectively agreed to donate biospecimens for research use [13, 14]. Factors such as gender, age, education level, and knowledge were mentioned to play a significant role in the willingness of donating [15, 16]. In addition, religious variances, cultural and social trends, and concerns about lack of confidentiality were considered as influential factors [16, 17]. Porteri et al. (2014) also found participants who showed more positive views on biomedical research to be in favour of donating biological samples for biobanking [13]. Domaradzki and Pawlikowski reviewed the existing research on public knowledge and donors’ views, perceptions, and attitudes on biobanking, and concluded that the public lacks knowledge about biobanking, yet their willingness for donation is high [17].

There are a few studies evaluating how the Saudi population would perceive the idea of donating their dental biospecimens [7, 16]. Although Saudis had poor knowledge about biobanking, sources of, and therapeutic uses of DSCs, they demonstrated positive attitudes toward participation in a future DSCs bank. More studies are needed to explore and understand the population’s perspectives in terms of willingness and concerns toward donating dental biospecimens in biorepositories for future research. Therefore, the aim of this study is to assess the willingness among adults attending outpatient clinics at King Abdulaziz Medical City (KAMC) to donate dental tissue samples in a biorepository for research purposes, and to identify the significant predictors for positive attitudes and willingness to donate dental biospecimens.

## Methods

### Study design and participants

This cross-sectional study was conducted at KAMC in Riyadh city, Saudi Arabia, after obtaining the ethical approval from the institutional review board of the National Guard Health Affairs, Riyadh, Saudi Arabia (RSS21R/017/07) and in accordance with relevant guidelines and regulations. Based on a previous study [7], where the expected prevalence of the willingness of the outpatients to participate in dental biorepository for research purposes was 70%, a sample size of 318 adults was needed to estimate the main outcome of this study with 5% margin of error at 95% level of confidence. To compensate for questionnaires with incomplete responses, a total sample of 401 adult subjects, who are eligible in KAMC of both sexes were the target sample for the present study. Participants were conveniently selected for this study while sitting in the waiting areas because of their engagement with the health service. Data was collected using an anonymous, manual, paper-based questionnaire distributed among adult Saudi patients attending King Saud bin Abdulaziz University for Health Sciences/College of dentistry (KSAU-HS/COD), Ambulatory Clinical Care (ACC) of KAMC, and King Abdullah Specialist Children Hospital (KASCH). Any subject fulfilling the inclusion criteria had the right not to participate in the study or withdraw during answering the questionnaire without completion.

### Data collection

The research team developed the questionnaire by referring to prior validated research [1, 4, 7, 16, 18]. Since the aim of this study could not be adequately addressed by a single existing research questionnaire in literature, a tailored questionnaire was employed based on combining previously published surveys [1, 4, 7, 16, 18], and enriching the questionnaire with additional questions to collect useful and related data. With the help of experts in King Abdullah International Medical Research Center (KAIMRC), the content validity was ensured using the judgmental approach to guarantee that the questionnaire is clear and relevant to the people residing in Saudi Arabia and attending KAMC. Additionally, test–retest reliability was performed in a pilot study of 15 subjects. The questionnaire focused on three main parts; the first part is about demographic, personal characteristics, and previous experience regarding biorepositories. This part includes age, gender, education, nationality, type of employment (medical or not), income, marital status, having children, residence, general health status, previous hospitalization, family medical history, and previous research participation and donation. The second part assessed the participant’s knowledge about biorepositories and its role in medical research using an 9-item scale. Whereas the last part of the survey included questions that evaluated the willingness and attitudes towards participating in donating dental biospecimens.

### Data analysis

The main outcomes in this study are participants’ attitudes and willingness to donate tissue to biobanks for biomedical research purposes and knowledge about biobanking. The willingness to donate was calculated as a binary variable (Yes/No) based on the respondent answers in Part II of the questionnaire (Are you willing to donate extracted teeth? (Yes/No), deciduous teeth? (Yes/No), excess surgical tissues? (Yes/No), saliva? (Yes/No) to be stored for future research purposes?). Willingness score was calculated by assigning a score of 0 for negative answers, and 1 for positive answers. Hence, the willingness score ranged from a minimum of 0 to a maximum of 4. Similarly, for each of the 9-item knowledge questions, a score of 1 was assigned when the participant gave a correct answer, and a score of 0 for an incorrect answer. The percentage of participants who gave a correct answer for each knowledge question was calculated. In addition, for each participant, a total knowledge score was calculated by summing across questions, with scores ranging from a minimum of 0 to a maximum of 9. To evaluate participants’ attitudes towards willingness to donate tissues to biobanks for biomedical research purposes, a 13-item attitude statement scale (5-point Likert scale) was used. Negative attitude statements scored from 1 (strongly agree) to 5 (strongly disagree) and the reverse was used for positive attitude statements. Accordingly, the minimum total score for attitude questions is 13 while the maximum is 65.

Categorical data was described using frequencies and percentages, whereas continuous data was expressed using means and standard deviations. The chi-squared test and Fishers Exact Test were used as tests of significance to compare categorical data, while Mann–Whitney test and Kruskal–Wallis test were used to compare numerical data. Multivariate analyses were performed with logistic regression models to determine significant predictors to willingness of the participant to donate surgical dental tissues. The choice of the variables in the model was based on the results of univariate analyses, where only the significant variables in these analyses were entered in the logistic regression analysis. For all statistical analyses, the significance level was set at 0.05 using Statistical Analysis System (SAS) v. 9.4 for data entry and data analysis. All data generated or analysed during this study are included in the results section and supplementary information section (see Additional files 1–4).

## Results

Four hundred and one subjects took part in this survey, their demographic, socioeconomic, health- and donation-related characteristics are presented in Table 1. Half of respondents (52%) were between 30 and 50 years of age, and 39% were under 30, while females represented 60% of the sample. Of the 401 participants, 194 (48%) had undergraduate university education, and only 5% received graduate or higher levels. Regarding the health status, 21% of the subjects reported being diagnosed with a chronic disease, and 46% reported previous hospitalization. Approximately 65% and 5% have had previous testing/donation of blood and tissues respectively, whereas only 57 (14%) participants have been involved in medical research.

**Table1:** Participants’ characteristics and their association with willingness to donate dental biospecimens

Variables	Frequency (%)	Willingness Score		*P* value
		0–2 n (%)	3–4 n (%)	
Total	401	144	257	
Age of the participant				
< 30	157 (39.15%)	59 (40.97%)	98 (38.13%)	
30–50	208 (51.87%)	70 (48.61%)	138 (53.70%)	0.5445
> 50	36 (8.98%)	15 (10.42%)	21 (8.17%)	
Gender of the participant				
Male	161 (40.15%)	69 (47.92%)	92 (35.80%)	0.0196*
Female	240 (59.85%)	75 (52.08%)	165 (64.20%)	
Nationality of the participant				
Saudi	397 (99.00%)	142 (98.61%)	255 (99.22%)	0.6206
Non-Saudi	4 (1.00%)	2 (1.39%)	2 (0.78%)	
Marital status of the participant				
Married	266 (66.33%)	94 (65.28%)	172 (66.93%)	
Single	117 (29.18%)	44 (30.56%)	73 (28.40%)	0.8962
Widowed/divorced	18 (4.49%)	6 (4.17%)	12 (4.67%)	
Education level completed by the participant				
High school or less	186 (46.38%)	82 (56.94%)	104 (40.47%)	
Undergraduate	194 (48.38%)	56 (38.89%)	138 (53.70%)	0.0065*
Graduate or more	21 (5.24%)	6 (4.17%)	15 (5.84%)	
Current employment status of the participant				
Employed	163 (40.65%)	55 (38.19%)	108 (42.02%)	
Unemployed	118 (29.43%)	45 (31.25%)	73 (28.40%)	
Student	64 (15.96%)	23 (15)	41 (15.95%)	0.4315
Retired	21 (5.24%)	11 (7.64)	10 (3.89%)	
Home duties	35 (8.73%)	10 (6.94%)	25 (9.73%)	
Type of employment of the participant				
Medical	42 (10.47%)	9 (6.25%)	33 (12.84%)	0.0418*
Non-medical	359 (89.53%)	135 (93.75%)	224 (87.16%)	
Monthly income of the participant				
< 5000SAR	106 (26.43%)	49 (34.03%)	57 (22.18%)	
5000–10,000SAR	167 (41.65%)	60 (41.67%)	107 (41.63%)	0.0111*
> 10,000SAR	128 (31.92%)	35 (24.31%)	93 (36.19%)	
Children of the participant				
Yes	262 (65.34%)	96 (66.67%)	166 (64.59%)	0.7430
No	139 (34.66%)	48 (33.33%)	91 (35.41%)	
Chronic diseases of the participant				
Yes	83 (20.70%)	35 (24.31%)	48 (18.68%)	0.1997
No	318 (79.30%)	109 (75.69%)	209 (81.32%)	
Previous hospitalization of the participant				
Yes	183 (45.64%)	64 (44.44%)	119 (46.30%)	0.7544
No	218 (54.36%)	80 (55.56%)	138 (53.70%)	
Previous blood testing and/or donation by the participant				
Yes	262 (65.34%)	82 (56.94%)	180 (70.04%)	
No	139 (34.66%)	62 (43.06%)	77 (29.96%)	0.0089*
Previous tissue (organ) testing and/or donation by the participant				
Yes	20 (4.99%)	3(2.08%)	17(6.61%)	
No	381(95.01%)	141(97.92%)	240(93.39%)	0.0551
Previous Involvement in medical research of the participant				
Yes	57 (14.21%)	9 (6.25%)	48 (18.68%)	
No	344 (85.79%)	135 (93.75%)	209 (81.32%)	0.0005*

*P < 0.05

**Fig. 1:**
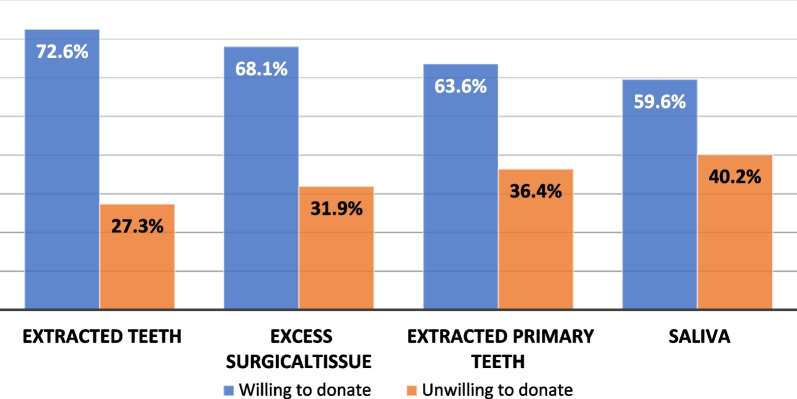
Sample preferences of the participants who are willing to donate dental biospecimens for biobanking and future research

The willingness to provide dental biological samples for research purposes was declared by about two-thirds (66%) of the participants who preferred to donate extracted teeth (n = 291, 72.6%), followed by excess surgical tissues (n = 273, 68.1%), extracted primary teeth (n = 255, 63.6%), and then donating saliva (n = 239, 59.6%) as shown in Fig. 1. Such large willingness was not modulated by health-related issues such as previous hospitalization or being diagnosed with chronic diseases, but by the subjects’ gender (*p* value = 0.0196), previous involvement in medical research (*p* value = 0.0005), previous blood testing or donation (*p* value = 0.0089), education (*p* value = 0.0065), monthly income (*p* value = 0.0111), and medical/nonmedical employment (*p* value = 0.0418) (Table 1). Regression analysis allowed the identification of the first three variables, namely gender, previous involvement in medical research, and previous blood testing or donation as strong predictors for increased willingness to donate dental biospecimens.

**Fig. 2:**
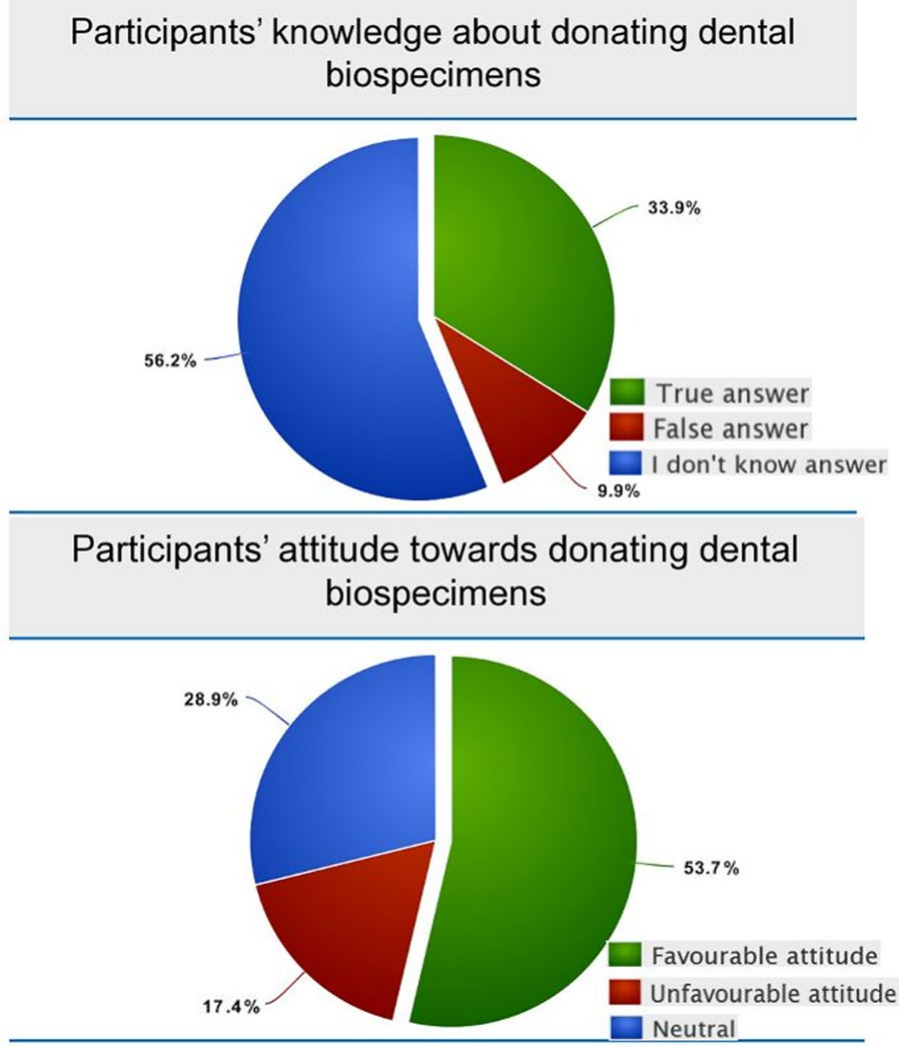
Participants’ knowledge about- and attitudes towards donating dental biospecimens to a repository

Although the majority of the respondents were willing to participate in dental biorepositories for research purposes, only 33.9% showed good level of knowledge while 56% of the participants answered the 9-item knowledge questions with the choice of “I don’t know”; in other words, they lacked the necessary knowledge (Fig. 2). Table 2 shows the participants’ responses to the biobanking knowledge questions. Only 13.7% of the participants have heard of the term Dental biobank or Dental biorepositories, whereas almost half of them (47.4%) knew the purpose of biobanks. Additionally, 33.2% were able to correctly define biospecimens as being samples and/or biomolecules with annotated clinical, socioeconomic and lifestyle data, but only 74 (18.5%) subjects believed that collecting stem cells is not an invasive procedure. When asked about the consent form, 243 subjects (60.1%) were aware that donating biospecimens to biorepositories is preceded by signing a consent form, likewise 44.1% knew that there is a standard operating procedure for biobanks, and 31.4% understood that their data would be kept confidential. Factorial analysis revealed that previous involvement in medical research (*p* value = 0.0220), education level (*p* value = 0.0002), type of employment (*p* value = 0.0137), and monthly income (*p* value = 0.0205) variables are significantly associated with knowledge about donating dental biospecimens for research purposes (Table 3).

**Table 2:** Participants’ knowledge about donating dental biospecimens

Items	True* n (%)	False** n (%)	I don’t know n (%)
I have heard about the term Dental biobank or Dental biorepositories	55 (13.72%)	107 (26.68%)	239 (59.60%)
Biospecimens are samples and/or biomolecules with annotated clinical, socioeconomic and lifestyle data	133 (33.17%)	20 (4.99%)	248 (61.85%)
Biobanks collects and stores biospecimens for research purposes	190 (47.38%)	13 (3.24%)	198 (49.38%)
Donating biospecimens requires signing a consent form	243 (60.60%)	19 (4.74%)	139 (34.66%)
Biospecimen data will be kept confidential and anonymous	126 (31.42%)	72 (17.96%)	203 (50.62%)
There is a standard operating procedure for biobanks to collect, process, store and release biospecimens	177 (44.14%)	13 (3.24%)	211 (52.62%)
Collecting stem cells is not an invasive procedure	74 (18.45%)	49 (12.22%)	278 (69.33%)
Stem cells could be collected from teeth and oral biospecimens	117 (29.18%)	32 (7.98%)	252 (62.84%)
Biospecimens from dental tissues can be used to treat many diseases	107 (26.68%)	34 (8.48%)	260 (64.84%)

*Correctly answered by the participant

**Incorrectly answered by the participant

**Table 3:** Association between participants’ characteristics and knowledge score

Variables	Knowledge score		*P* value
	0–4 n (%)	5–9 n (%)	
Total	272 (67.83%)	129 (32.17%)	
Age of the participant			
< 30	106 (38.97%)	51 (39.53%)	
30–50	142 (52.21%)	66 (51.16%)	0.9608
> 50	24 (8.82%)	12 (9.30%)	
Gender of the participant			
Male	113 (41.54%)	48 (37.21%)	0.4460
Female	159 (58.46%)	81 (62.79%)	
Nationality of the participant			
Saudi	269 (98.90%)	128 (99.22%)	1.0000
Non-Saudi	3 (1.10%)	1 (0.78%)	
Marital status of the participant			
Married	184 (67.65%)	82 (63.57%)	
Single	76 (27.94%)	41 (31.78%)	0.7084
Widowed/divorced	12 (4.41%)	6 (4.65%)	
Education level completed by the participant			
High school or less	144 (52.94%)	42 (32.56%)	
Undergraduate	118 (43.38%)	76 (58.91%)	0.0002*
Graduate or more	10 (3.68%)	11 (8.53%)	
Current employment status of the participant			
Employed	105 (38.60%)	58 (44.96%)	
Unemployed	83 (30.51%)	35 (27.13%)	
Student	44 (16.18%)	20 (15.50%)	0.8354
Retired	15 (5.51%)	6 (4.65%)	
Home duties	25 (9.19%)	10 (7.75%)	
Type of employment of the participant			
Medical	21 (7.72%)	21 (16.28%)	0.0137*
Non-medical	251 (92.28%)	108 (83.72%)	
Monthly income of the participant			
< 5000SAR	81 (29.78%)	25 (19.38%)	
5000–10,000SAR	115 (42.28%)	52 (40.31%)	0.0205*
> 10,000SAR	76 (27.94%)	52 (40.31%)	
Children of the participant			
Yes	178 (65.44%)	84 (65.12%)	
No	94 (34.56%)	45 (34.88%)	1.0000
Chronic diseases of the participant			
Yes	59 (21.69%)	24 (18.60%)	
No	213 (78.31%)	105 (81.40%)	0.5118
Previous hospitalization of the participant			
Yes	126 (46.32%)	57 (44.19%)	
No	146 (53.68%)	72 (55.81%)	0.7477
Previous blood testing and/or donation by the participant			
Yes	177 (65.07%)	85 (65.89%)	
No	95 (34.93%)	44 (34.11%)	0.9109
Previous tissue (organ) testing and/or donation by the participant			
Yes	13 (4.78%)	7 (5.43%)	
No	259 (95.22%)	122 (94.57%)	0.8080
Previous Involvement in medical research of the participant			
Yes	31 (11.40%)	26 (20.16%)	
No	241 (88.60%)	103 (79.84%)	0.0220*

*P < 0.05

**Fig. 3:**
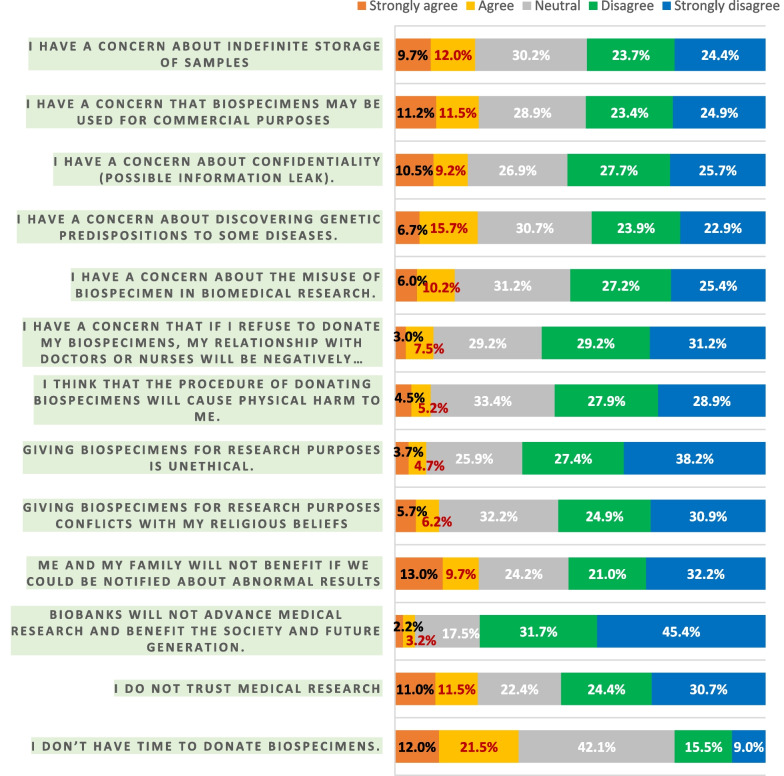
Participants’ attitude towards donating dental biospecimens. The agree and strongly agree categories represent the negative (unfavorable) attitude while disagree and strongly disagree categories represent the positive (favorable) attitude

Despite the notable lack of knowledge, 54% of the studied population had favorable attitudes towards donating dental biospecimens, and only 17% were negative while the remaining 29% were neutral (Fig. 2). The associations between different variables and this attitude are summarized in Table 4. Female participants with higher level of education and higher monthly income showed significantly higher attitude scores as compared to their counter groups with *p* value s equal to 0.0273, 0.0024, and 0.0004 respectively. Figure 3 shows the response of our participants to 13 attitude statements describing factors that may influence the decision of participants to donate biospecimens. For the participants who had negative attitudes, having no time to donate (33.5%) was the most frequently reported reason for their decision not to donate, followed by concern that biospecimens may be used for commercial purposes (22.7%), not trusting medical research (22.5%), fear of discovering genetic predispositions (22.4%), and not benefiting them and their families (21.7%). On the other hand, the primary recorded factor for favorable attitude towards donating dental biospecimens to biobanks was that donations would advance medical research and benefit society and future generation (77.1%). Other factors included participants beliefs that donation is not unethical (65.6%), and that they have no concerns about confidentiality (53.4%).

**Table 4:** Association between participants’ characteristics and attitude score

Variables	Attitude score		*P* value
	< 30	30–65	
	n (%)	n (%)	
Total	152 (37.91%)	249 (62.09%)	
Age of the participant			
< 30	58 (38.16%)	99 (39.76%)	
30–50	79 (51.97%)	129 (51.81%)	0.8731
> 50	15 (9.87%)	21 (8.43%)	
Gender of the participant			
Male	72 (47.37%)	89 (35.74%)	0.0273*
Female	80 (52.63%)	160 (64.26%)	
Nationality of the participant			
Saudi	152 (100.00%)	245 (98.39%)	0.3021
Non-Saudi	0 (0.00%)	41 (61%)	
Marital status of the participant			
Married	108 (71.05%)	158 (63.45%)	
Single	39 (25.66%)	78 (31.33%)	0.2796
Widowed/divorced	5 (3.29%)	13 (5.22%)	
Education level completed by the participant			
High school or less	87 57.24%)	99 (39.76%)	
Undergraduate	60 (39.47%)	134 (53.82%)	0.0024*
Graduate or more	5 (3.29%)	16 (6.43%)	
Current employment status of the participant			
Employed	56 (36.84%)	107 (42.97%)	
Unemployed	45 (29.61%)	73 (29.32%)	
Student	28 (18.42%)	36 (14.46%)	0.7064
Retired	9 (5.92%)	12 (4.82%)	
Home duties	14 (9.21%)	21 (8.43%)	
Type of employment of the participant			
Medical	13 (8.55%)	29 (11.65%)	0.4014
Non-medical	139 (91.45%)	220 (88.35%)	
Monthly income of the participant			
< 5000SAR	45 (29.61%)	61 (24.50%)	
5000–10,000SAR	76 (50.00%)	91 (36.55%)	0.0004*
> 10,000SAR	31 (20.39%)	97 (38.96%)	
Children of the participant			
Yes	102 (67.11%)	160 (64.26%)	0.5898
No	50 (32.89%)	89 (35.74%)	
Chronic diseases of the participant			
Yes	30 (19.74%)	53 (21.29%)	0.7996
No	122 (80.26%)	196 (78.71%)	
Previous hospitalization of the participant			
Yes	67 (44.08%)	116 (46.59%)	0.6796
No	85 (55.92%)	133 (53.41%)	
Previous blood testing and/or donation by the participant			
Yes	94 (61.84%)	168 (67.47%)	0.2798
No	58 (38.16%)	81 (32.53%)	
Previous tissue (organ) testing and/or donation by the participant			
Yes	6 (3.95%)	14 (5.62%)	0.6371
No	146 (96.05%)	235 (94.38%)	
Previous Involvement in medical research of the participant			
Yes	15 (9.87%)	42 (16.87%)	
No	137 (90.13%)	207 (83.13%)	0.0560

*P < 0.05

## Discussion

The potential of DSCs-based regenerative therapy in both fields of medicine and dentistry has triggered/attracted many biomedical researchers to investigate these cells intensely. DSCs, like other MSCs, are characterized with self-renewal capacity and multidifferentiation potential. Additionally, they are easily accessible and prevail long-life. DSCs can be isolated from several tissues in the oral cavity such as primary teeth, pulpal tissue, periodontal ligaments, and apical papilla, and then cryopreserved in biobanks/repositories for future research or therapeutic uses. Establishing such biobanks is largely dependent on public’s attitudes and willingness to donate their biosamples, therefore, a suitable assessment is essential to understand their attitudes and baseline knowledge, and to shed some light on the factors that have major influence on their willingness for donation.

Sixty-six percent of participants were willing to provide dental/oral biological samples for biobanks. This percentage is within the range registered in previous attitude surveys, which oscillated between 56 and 91% [1, 4, 7, 13, 16–23]. The level of willingness reported in this study (66%) is consistent with that found in two local studies, where 61% of the Saudi public would like to contribute to teeth biobanks, while 70% were willing to allow their excess surgical tissue to be used in research [7, 19]. Similarly, 63%, 64%, 71% and 75% of the respondents from Egyptian, Jordanian, Swedish and UK public, respectively, agreed with donation [20–23]. Another Jordanian study showed higher level of willingness to donate biospecimens for future research (79%), however, an informational paragraph about participation in biobanking was added in the questionnaire [1]. Education also acted as a modulator that increased the willingness of Italian public to donate biological samples to 86% [13]. Notably, local studies demonstrated higher enthusiasm to donate biospecimens to biobanks by Saudi dental practitioners (91%) and senior healthcare students (89%). Both percentages are among the highest registered in the biospecimens donation era, possibly because the participants were professionals in health care facilities and students in academic institutions [7, 16].

Our results showed that subjects’ prior participation in medical research, blood testing/donation, and gender can predict their willingness towards donating dental biospecimens (Table 1). In previous reports, participation in biomedical, genetic, and pharmacogenomics research has also been found to positively influence the Italian, Swedish, and Japanese publics [13, 22, 24]. Such significant association was not reported by Merdad et al. who targeted senior health care students instead of the public [16]. In line with Aljumah et al. (2011) studies who surveyed the same public group (KAMC outpatients), we found that previous blood testing is a significant predictor for higher willingness scores [18, 19]. In consistency with this study’s result that gender is a strong predictor, Saudi and Egyptian females were also found to be more willing to donate to biobanks than males [19, 20]. However, Domaradzki and Pawlikowski mentioned in their review that various studies revealed that male participants were more eager to donate [17]. Additionally, individuals with higher education degrees, higher income, and/or work in a medical facility were significantly more willing to donate their excess surgical tissues and dental samples for future biomedical research than other participants (Table 1). As found in other surveys, the willingness to participate by biosample donations was modulated by the years of schooling, confirming the relation that the higher the education level, the more the confidence in science [1, 13, 17, 19, 21, 25]. Likewise, people with higher economic status significantly expressed a more positive view toward donation [17, 26]. However, other reports suggest that not only education and income factors but also individual perception and possibly knowledge may affect someone’s choice [13]. Other factors such as age, history of previous hospitalization, and having chronic diseases were identified as predictors of willingness to donate biospecimens, but not found significant in our study [1, 17, 19]. Considering age, the most controversial factor, several studies found that not only older participants, but also middle-aged subjects (40–65 years) are in favor of donation more than younger people [17, 22, 27, 28]. On the other hand, some studies suggest that as age increases, the total number of individuals who are willing to donate decreases, whereas in the present survey, age was not a modulating factor [17, 21]. This inconsistency between the aforementioned reports could be attributed to social and cultural variances among different populations.

Regarding participants’ preferences to donate specific dental specimens, the highest willingness rate was recorded for teeth (73%), followed by excess surgical tissues (68%) and then primary teeth (64%) (Fig. 1). These rates are very close to the reported 70–71% of Saudi and Swedish populations who were willing to donate their surplus surgical tissues for research purposes [19, 22], and is in agreement with Hassona et al. study (2016) where excess tissue and extracted teeth had higher reported rates to be donated [4]. When body fluids were compared, the willingness to donate saliva was higher than that of urine, but less than blood donations, possibly due to familiarity of the blood donation process [16]. In our survey, saliva was the least preferred to be donated, as only 60% of the respondents were willing to donate this type of sample and the proportion was as low as 19% in another report [4]. Being unfamiliar with the concept of donating saliva or feeling embarrassed to give a saliva sample may explain these relatively low willingness scores.

Paradoxically, two-thirds of (66%) of our respondents were willing to participate in dental biorepositories for research purposes, while another finding revealed that 66% of our sample showed insufficient level of knowledge about biobanking and biospecimens. Out of these, 56% answered the 9-item knowledge questions with an “I don’t know” choice, while 10% selected “false” (Fig. 2). Despite the deficits in knowledge of the studied public, most research demonstrated positive views on biobanking, for instance, 77% of Finns expressed positive attitudes toward creating a local biobank, while 83% of them showed little knowledge about biobanking [29]. Similarly, two-thirds of Europeans and Americans have not heard about biobanks [17, 30]. Rahm  et al., who distributed brochures prior to conducting their survey, reported that 85% of participants were able to choose the correct answers regarding biospecimens and research [31]. As such, the critical role of educational health campaigns that raise public awareness about the importance of dental biospecimens’ donation for biobanking and for biomedical research is highly emphasized [7].

A person’s attitude is invisible, inaccessible, and unperceivable, yet the magnitude of this attitude is measurable by assessing the reaction or responses to a situation, person, event, etc.[32]. Attitudes toward donating dental biospecimens were evaluated in this study by using 13 statements addressing the potential benefits/harms, concerns, and ethics of biobanking (Fig. 3). We found 54% of participants hold favorable views of donation, while only 17% expressed negative attitudes. Such findings are in concordance with other several national, regional, and international studies [17]. However, it is noteworthy that some individuals may express positive views toward donation in general, but only a few would agree to donate their own samples [19]. The worldwide public responses to biobank participation are largely positive in general, but simultaneously, there are a number of concerns and reservations expressed by the publics [12].

In our study, many respondents (33.5%) were discouraged by having inadequate time to undergo the donation procedure, similarly, 44% of Jordanians have concerns with the time factor [1]. Although fear of having not enough time was identified as a main reason for deterring biospecimen donations in our survey, it seems that the time factor did not completely deter 24.5% of our participants from contributing to biobanks, however, time could be an influential factor that modulates their decision on whether to participate or not. On the other hand, as declared by 77% of our respondents, the most important motivation found was “biobanks will advance medical research and benefit society and future generations”, and only 5.4% believed the opposite view. Domaradzki and Pawlikowski stated in their review that helping future generations, generating new knowledge, and developing new therapies are major factors influencing the willingness to donate biospecimens [17]. In Sweden, 89% of respondents were strongly motivated by the idea that biobank research would benefit future patients [22], and 74% of Americans would donate to support scientific research [31]. Likewise, the major reason for 76% of Italian respondents was utilization of research facilitated by biobanks to discover novel therapies [13]. Feeling obliged to comply with healthcare personnel could be motivated by patients’ fears that when declining the request to donate biospecimens, the quality of health care provided would be affected. On the contrary, such fears were expressed by 1% of Swedish [22], 19.3% of Saudis [18, 19], and only 10.5% of our population sample. Similarly, only few participants (9–13%) believed that the procedure of donating dental biospecimens is not physically harmful nor is it conflicting with their religious and ethical values. Nevertheless, approximately 20–23% of the surveyed subjects justified their negative attitudes toward specimen’s donation with concerns about confidentiality, trusting medical research, discovering genetic predispositions to some diseases, biospecimens use for commercial purposes, and indefinite storage of samples. These findings agree with previous studies, which concluded that trusting biobanks and medical research correlates positively with favorable views on biosamples’ donation [17, 20, 29, 30]. Other reports emphasized the importance of building trust with the public, since concerns about confidentiality were found to be a major reason for not being a donor [17, 28, 31]. Apart from these concerns, measuring general attitudes towards biobanks and/or biomedical research revealed most participants believed that the benefits outweigh the risks.

We acknowledge that the present study had some limitations; firstly, the participants surveyed were recruited in an outpatient medical department of one governmental sector. Secondly, the study was conducted in one city only, Riyadh (the capital). As it is possible that our results may not represent the attitudes of the general public of Saudi Arabia, further studies employing a sample population that covers the whole nation are recommended. Thirdly, we evaluated the future intention to donate dental biospecimens to biobanks, which is regarded as hypothetical and may not reflect actual behavior. These circumstances could influence our participants’ answers and may not be considered as representative of the public of Saudi Arabia. More studies on factors that enhance and contribute to increased willingness for participating to dental biobanks are recommended. Finally, to enhance the public knowledge and eventually encourage dental biospecimens donation for future research, it is recommended to plan for awareness campaigns and educational programmes.

## Conclusions

The current study was designed to assess the willingness among the attendants of outpatient clinics to donate dental specimens to biobanks for research purposes, and to identify the significant predictors for positive attitudes toward donation. The results indicated the following:

- Although the majority of participants exhibited lack of knowledge about dental biorepositories, they showed very high willingness and positive attitude towards donating dental biospecimens. This favorable attitude is, in turn, encouraging for the future establishment of dental biorepositories in Saudi Arabia.
- Factors associated with strong willingness to donate were completion of higher education, employment in medical field, high monthly income, female gender, previous blood testing or donation, and prior involvement in medical research. The later three are significant predictors.

## Supplementary Information

**Additional file 1.** Frequency distribution of participants’ answers of the questionnaire which consisted of three main parts: personal characteristics, knowledge about biorepositories, and attitudes toward donating dental bio specimens.

**Additional file 2.** Descriptive analysis of willing score.

**Additional file 3.** Descriptive analysis of knowledge score.

**Additional file 4.** Descriptive analysis of attitude score.

## Data Availability

All data generated or analysed during this study are included in this published article and its supplementary information files.

## Abbreviation

DNA: Deoxyribonucleic acid
MSCs: Mesenchymal stem cells
DSCs: Dental stem cells
KAMC: King Abdulaziz Medical City
IRB: Institutional Review Board
KSAU-HS/COD: King Saud Bin Abdulaziz University for Health Sciences/College of Dentistry
ACC: Ambulatory Clinical Care
KASCH: King Abdullah Specialist Children Hospital
KAIMRC: King Abdullah International Medical Research Center
SAS: Statistical Analysis System
