# Supplementary material for: Assessment of willingness of Saudi public to participate in a dental biorepository for research purposes
Source: BMC Oral Health. 2023 Feb 7;23:80. doi: 10.1186/s12903-023-02775-9 (PMC9906834; doi:10.1186/s12903-023-02775-9)
Supplement: Supplementary file 1 — Additional file 1. Frequency distribution of participants’ answers of the questionnaire which consisted of three main parts: personal characteristics, knowledge about biorepositories, and attitudes toward donating dental bio specimens. [file 12903_2023_2775_MOESM1_ESM.rtf]

Additional file 1Appendix 1: Frequency distribution of participants’ answers of the questionnaire which consisted of three main parts: personal characteristics, knowledge about biorepositories, and attitudes toward donating dental bio specimens Where responses were collected			
clinic	Frequency	Percent	
Dental	157	39.15	
Non-dental	244	60.85	
Age of the participant			
Age	Frequency	Percent	
30 or less	157	39.15	
30-50	208	51.87	
More than 50	36	8.98	
Gender of the participant			
Gender	Frequency	Percent	
Male	161	40.15	
Female	240	59.85	
Nationality of the participant			
Nationality	Frequency	Percent	
Saudi	397	99.00	
Non-Saudi	4	1.00	
Marital status of the participant			
_Marital_status	Frequency	Percent	
Married	266	66.33	
Single	117	29.18	
Widowed/Divorced	18	4.49	
Education level completed by the participant			
Educational_level_completed	Frequency	Percent	
High school or less	186	46.38	
Undergraduate	194	48.38	
Graduate or more	21	5.24	
Current employment status of the participant			
Current_employment_status	Frequency	Percent	
Employed	163	40.65	
Unemployed	118	29.43	
Student	64	15.96	
Retired	21	5.24	
Home duties	34	8.48	
15	1	0.25	
Type of employment of the participant			
_Type_of_employment	Frequency	Percent	
Medical	42	10.47	
Non-medical	358	89.28	
4	1	0.25	
Monthly income of the participant			
_Which_of_these_describe_your_mo	Frequency	Percent	
Less than 5000SAR	106	26.43	
5000-10000SAR	167	41.65	
More than 10000SAR	128	31.92	
children of the participant			
Do_you_have_any_children_	Frequency	Percent	
Yes	262	65.34	
No	137	34.16	
3	2	0.50	
Chronic diseases of the participant			
Do_you_have_any_chronic_diseases	Frequency	Percent	
Yes	83	20.70	
No	318	79.30	
Previous hospitalization of the participant			
Previous_hospitalization_	Frequency	Percent	
Yes	183	45.64	
No	218	54.36	
Previous blood testing and/or donation by the participant by the participant  by the participant			
Previous_blood_testing_and_or_do	Frequency	Percent	
Yes	261	65.09	
No	139	34.66	
3	1	0.25	
Previous tissue (organ) testing and/or donation by the participant			
Previous_tissue__organ__testing	Frequency	Percent	
Yes	20	4.99	
No	380	94.76	
3	1	0.25	
Previous Involvement in medical research of the participant			
Previous_Involvement_in_medical	Frequency	Percent	
Yes	57	14.21	
No	342	85.29	
3	2	0.50	
I have heard about the term Dental biobank or Dental biorepositories.			
I_have_heard_about_the_term_Dent	Frequency	Percent	
True	55	13.72	
False	107	26.68	
I don't know	239	59.60	
Biospecimens are samples and/or biomolecules with annotated clinical, socioeconomic and lifestyle data.			
Biospecimens_are_samples_and_or	Frequency	Percent	
True	133	33.17	
False	20	4.99	
I don't know	248	61.85	
Biobanks collects and stores biospecimens for research purposes.			
Biobanks_collects_and_stores_bio	Frequency	Percent	
True	190	47.50	
False	13	3.25	
I don't know	196	49.00	
33	1	0.25	
Frequency Missing = 1			
Donating biospecimens requires signing a consent form.			
Donating_biospecimens_requires_s	Frequency	Percent	
True	243	60.60	
False	19	4.74	
I don't know	139	34.66	
Biospecimen data will be kept confidential and anonymous			
Biospecimen_data_will_be_kept_co	Frequency	Percent	
True	126	31.42	
False	72	17.96	
I don't know	203	50.62	
There is a standard operating procedure for biobanks to collect, process, store and release biospecimens.			
There_is_a_standard_operating_pr	Frequency	Percent	
True	177	44.14	
False	13	3.24	
I don't know	211	52.62	
Collecting stem cells is an invasive procedure.			
Collecting_stem_cells_is_an_inva	Frequency	Percent	
True	74	18.45	
False	49	12.22	
I don't know	278	69.33	
Stem cells could be collected from teeth and oral biospecimens.			
Stem_cells_could_be_collected_fr	Frequency	Percent	
True	117	29.18	
False	32	7.98	
I don't know	252	62.84	
Biospecimens from dental tissues can be used to treat many diseases.			
Biospecimens_from_dental_tissues	Frequency	Percent	
True	107	26.68	
False	34	8.48	
I don't know	260	64.84	
Participant willingness to donating biological samples			
Are_you_willing_to_donate_biolog	Frequency	Percent	
Yes	176	43.89	
No	218	54.36	
3	7	1.75	
Participant willingness to donate child's teeth			
If_you_are_a_parent_guardian_are	Frequency	Percent	
Yes	165	41.15	
No	123	30.67	
3	113	28.18	
Are you willing to donate extracted teeth?			
Are_you_willing_to_donate_extrac	Frequency	Percent	
Yes	290	72.32	
No	110	27.43	
3	1	0.25	
Are you willing to donate extracted primary teeth?			
Are_you_willing_to_donate_extra1	Frequency	Percent	
Yes	252	62.84	
No	146	36.41	
3	3	0.75	
Are you willing to donate excess tissue			
Are_you_willing_to_donate_excess	Frequency	Percent	
Yes	268	66.83	
No	128	31.92	
3	1	0.25	
4	4	1.00	
Are you willing to donate saliva			
Are_you_willing_to_donate_saliva	Frequency	Percent	
Yes	236	58.85	
No	160	39.90	
3	2	0.50	
4	3	0.75	
I don’t have time to donate biospecimens.			
I_don_t_have_time_to_donate_bios	Frequency	Percent	
Strongly agree	48	11.97	
Agree	86	21.45	
Neutral	169	42.14	
Disagree	62	15.46	
Strongly disagree	36	8.98	
I do not trust medical research.			
I_do_not_trust_medical_research_	Frequency	Percent	
Strongly agree	44	10.97	
Agree	46	11.47	
Neutral	90	22.44	
Disagree	98	24.44	
Strongly disagree	123	30.67	
Biobanks will advance medical research and benefit the society and future generation.			
Biobanks_will_advance_medical_re	Frequency	Percent	
Strongly agree	9	2.24	
Agree	13	3.24	
Neutral	70	17.46	
Disagree	127	31.67	
Strongly disagree	182	45.39	
Me and my family will benefit if we could be notified about abnormal results.			
Me_and_my_family_will_benefit_if	Frequency	Percent	
Strongly agree	52	12.97	
Agree	39	9.73	
Neutral	97	24.19	
Disagree	84	20.95	
Strongly disagree	129	32.17	
Giving biospecimens for research purposes conflicts with my religious beliefs.			
Giving_biospecimens_for_research	Frequency	Percent	
Strongly agree	23	5.74	
Agree	25	6.23	
Neutral	129	32.17	
Disagree	100	24.94	
Strongly disagree	124	30.92	
Giving biospecimens for research purposes is unethical.			
Giving_biospecimens_for_researc1	Frequency	Percent	
Strongly agree	15	3.74	
Agree	19	4.74	
Neutral	104	25.94	
Disagree	110	27.43	
Strongly disagree	153	38.15	
I think that the procedure of donating biospecimens will cause physical harm to me.			
I_think_that_the_procedure_of_do	Frequency	Percent	
Strongly agree	18	4.49	
Agree	21	5.24	
Neutral	134	33.42	
Disagree	112	27.93	
Strongly disagree	116	28.93	
I have a concern that if I refuse to donate my biospecimens that it will negatively affect my relationship with doctors or nurses which may affect the health care provided to me.			
I_have_a_concern_that_if_I_refus	Frequency	Percent	
Strongly agree	12	2.99	
Agree	30	7.48	
Neutral	117	29.18	
Disagree	117	29.18	
Strongly disagree	125	31.17	
I have a concern about the misuse of biospecimen in biomedical research.			
I_have_a_concern_about_the_misus	Frequency	Percent	
Strongly agree	24	5.99	
Agree	41	10.22	
Neutral	125	31.17	
Disagree	109	27.18	
Strongly disagree	102	25.44	
I have a concern about discovering genetic predispositions to some diseases.			
I_have_a_concern_about_discoveri	Frequency	Percent	
Strongly agree	27	6.73	
Agree	63	15.71	
Neutral	123	30.67	
Disagree	96	23.94	
Strongly disagree	92	22.94	
I have a concern about confidentiality (Possible information leak).			
I_have_a_concern_about_confident	Frequency	Percent	
Strongly agree	42	10.47	
Agree	37	9.23	
Neutral	108	26.93	
Disagree	111	27.68	
Strongly disagree	103	25.69	
I have a concern that biospecimens may be used for commercial purposes			
I_have_a_concern_that_biospecime	Frequency	Percent	
Strongly agree	45	11.22	
Agree	46	11.47	
Neutral	116	28.93	
Disagree	94	23.44	
Strongly disagree	100	24.94	
I have a concern about Indefinite storage of samples.			
I_have_a_concern_about_Indefinit	Frequency	Percent	
Strongly agree	39	9.73	
Agree	48	11.97	
Neutral	121	30.17	
Disagree	95	23.69	
Strongly disagree	98	24.44	
