# Supplementary material for: Assessment of willingness of Saudi public to participate in a dental biorepository for research purposes
Source: BMC Oral Health. 2023 Feb 7;23:80. doi: 10.1186/s12903-023-02775-9 (PMC9906834; doi:10.1186/s12903-023-02775-9)
Supplement: Supplementary file 2 — Additional file 2. Descriptive analysis of willing score. [file 12903_2023_2775_MOESM2_ESM.rtf]

Additional file 2. Appendix 2: Descriptive analysis of willing score.WillingScore	Frequency	Percent	
0	88	21.95	
1	18	4.49	
2	38	9.48	
3	63	15.71	
4	194	48.38	
Are you willing to donate extracted teeth?			
Are_you_willing_to_donate_extrac	Frequency	Percent	
Yes	291	72.57	
No	110	27.43	
Are you willing to donate extracted primary teeth?			
Are_you_willing_to_donate_extra1	Frequency	Percent	
Yes	255	63.59	
No	146	36.41	
Are you willing to donate excess tissue			
Are_you_willing_to_donate_excess	Frequency	Percent	
Yes	273	68.08	
No	128	31.92	
Are you willing to donate saliva								
Are_you_willing_to_donate_saliva	Frequency	Percent						
Yes	240	59.85						
No	161	40.15						
Analysis Variable : willingScore								
N	Mean	Std Dev	Median	Quartile Range	N Miss	Minimum	Maximum	
401	2.64	1.62	3.00	3.00	0	0.00	4.00	
