# Supplementary material for: Assessment of willingness of Saudi public to participate in a dental biorepository for research purposes
Source: BMC Oral Health. 2023 Feb 7;23:80. doi: 10.1186/s12903-023-02775-9 (PMC9906834; doi:10.1186/s12903-023-02775-9)
Supplement: Supplementary file 3 — Additional file 3. Descriptive analysis of knowledge score. [file 12903_2023_2775_MOESM3_ESM.rtf]

Additional file 3. Appendix 3: Descriptive analysis of knowledge score.knowledgeScore	Frequency	Percent	
0	101	25.19	
1	42	10.47	
2	34	8.48	
3	48	11.97	
4	47	11.72	
5	52	12.97	
6	38	9.48	
7	23	5.74	
8	13	3.24	
9	3	0.75	
								
								
								
								
Frequency Missing = 260								
Analysis Variable : knowledgeScore								
N	Mean	Std Dev	Median	Quartile Range	N Miss	Minimum	Maximum	
401	3.05	2.50	3.00	5.00	0	0.00	9.00	
