# Supplementary material for: Assessment of willingness of Saudi public to participate in a dental biorepository for research purposes
Source: BMC Oral Health. 2023 Feb 7;23:80. doi: 10.1186/s12903-023-02775-9 (PMC9906834; doi:10.1186/s12903-023-02775-9)
Supplement: Supplementary file 4 — Additional file 4. Descriptive analysis of attitude score. [file 12903_2023_2775_MOESM4_ESM.rtf]

Additional file 4. Appendix 4: Descriptive analysis of attitude score.attitudeScore	Frequency	Percent	
13	4	1.00	
14	2	0.50	
15	8	2.00	
16	4	1.00	
17	8	2.00	
18	10	2.49	
19	11	2.74	
20	6	1.50	
21	13	3.24	
22	9	2.24	
23	14	3.49	
24	12	2.99	
25	8	2.00	
26	13	3.24	
27	17	4.24	
28	13	3.24	
29	15	3.74	
30	14	3.49	
31	11	2.74	
32	23	5.74	
33	13	3.24	
34	16	3.99	
35	12	2.99	
36	11	2.74	
37	19	4.74	
38	11	2.74	
39	31	7.73	
40	11	2.74	
41	10	2.49	
42	9	2.24	
43	8	2.00	
44	7	1.75	
45	3	0.75	
46	2	0.50	
47	7	1.75	
48	3	0.75	
49	1	0.25	
50	3	0.75	
51	2	0.50	
52	2	0.50	
54	1	0.25	
56	1	0.25	
57	3	0.75	
								
								
								
								
								
								
								
Analysis Variable : attitudeScore								
N	Mean	Std Dev	Median	Quartile Range	N Miss	Minimum	Maximum	
401	31.59	9.22	32.00	15.00	0	13.00	57.00	
